# Analysis of Muscle Lipidome in Juvenile Rainbow Trout Fed Rapeseed Oil and Cochayuyo Meal

**DOI:** 10.3390/biom12060805

**Published:** 2022-06-09

**Authors:** John Quiñones, Rommy Díaz, Jorge F. Beltrán, Lidiana Velazquez, David Cancino, Erwin Muñoz, Patricio Dantagnan, Adrián Hernández, Néstor Sepúlveda, Jorge G. Farías

**Affiliations:** 1 Facultad de Ciencias Agropecuarias y Forestales, Universidad de La Frontera, Temuco 4780000, Chile; john.quinones@ufrontera.cl (J.Q.); rommy.diaz.pe@ufrontera.cl (R.D.); nestor.sepulveda@ufrontera.cl (N.S.); 2 Departamento de Ingeniería Química, Facultad de Ingeniería y Ciencias, Universidad de La Frontera, Temuco 4780000, Chile; beltran.lissabet.jf@gmail.com; 3 Programa de Doctorado en Ciencias Agroalimentarias y Medioambiente, Universidad de La Frontera, Temuco 4780000, Chile; l.velazquez01@ufromail.cl; 4 Escuela de Medicina Veterinaria, Facultad de Ciencias, Universidad Mayor, Temuco 4780000, Chile; cancinobaier@gmail.com; 5 Programa de Doctorado en Ciencias Mención Biología Celular y Molecular Aplicada, Universidad de La Frontera, Temuco 4780000, Chile; e.munoz09@ufromail.cl; 6 Núcleo de Investigación de Producción Alimentaria, Departamento de Ciencias Agropecuarias y Acuícolas, Facultad de Recursos Naturales, Universidad Católica de Temuco, Temuco 4780000, Chile; dantagna@uct.cl (P.D.); ajhernandez@uct.cl (A.H.); 7 Centro de Tecnología e Innovación de la Carne, Universidad de La Frontera, Temuco 4780000, Chile

**Keywords:** aquaculture, phospholipids, LC-MS/MS, seaweed, metabolomics, sustainability

## Abstract

This study aimed to analyze the effects on the lipidome of juvenile *Oncorhynchus mykiss* muscle fed 90% *Brassica napus* “rapeseed” oil and different amounts of *Durvillaea antarctica* “Cochayuyo” meal (1.5, 3 and 6%) as a replacement for cellulose. The analysis allowed for the identification of 329 lipids, mainly represented by phospholipids and fatty esters. The inclusion of *Brassica napus* oil significantly increased the levels of C18:2 species and fatty esters of hydroxylated fatty acids, which could play a bioactive role in human health. One of the most abundant lipids in all fillets was Phosphatidylcholine 33:6, which, according to the literature, could be considered a biomarker for the identification of *Oncorhynchus mykiss*. In all experimental diets, the species Phosphatidylethanolamine 15:1-18:24 showed four-fold higher levels than the control; increments of n-3- and n-6-rich phospholipids were also observed. Diets containing *Durvillaea antarctica* meal did not generate more significant variation in fish muscle phospholipids relative to the muscle of the rapeseed-oil-only group. These lipid species consist of medium- and long-chain fatty acids with different degrees of unsaturation. Still, it appears that the rapeseed oil masks the lipid contribution of the meal, possibly due to the low levels of total lipids in the macroalgae.

## 1. Introduction

Health specialists recommend regular fish consumption because fish tissues contain very long chain fatty acids (VLC-FA), which are mainly classified as omega-3 (n-3) fatty acids due to the position of the first double bond at carbon 3 in the opposite direction to the carboxyl group, the main omega-3 found in fish eicosapentaenoic acid (EPA) and docosahexaenoic acid (DHA) [1,2]. In recent years, fish production has reduced its dependence on marine fisheries and focused on aquaculture development [3]. Besides tilapia (*Oreochromis niloticus*) and salmon (*Salmo salar*), rainbow trout (*Oncorhynchus mykiss*) is one of the most important species in world aquaculture, with a fillet containing approximately 2500 mg/100 g of n-3, an n-3/n-6 ratio greater than 2, and a thrombogenic index and atherogenic index less than 0.5, which is highly beneficial for human health [4,5,6].

One of the significant challenges in aquaculture is replacing the fish oil used for aquafeed production, which is vital for proper development and growth. However, its high demand has made it a scarce and expensive ingredient. Rapeseed (*Brassica napus*) oil is an edible oil containing high levels of C18:1 and C18:2, and its inclusion in rainbow trout feed has no detrimental effects on fish growth and development, but an analysis of the fatty acid profile by gas chromatography indicates that the total n-3 levels of the fillet are significantly reduced [7,8,9]. However, it has been observed that dietary supplementation of macroalgae can improve the nutritional quality of fish. For example, a study evaluated the use of *Macrocystis pyrifera* in trout feed, showing that the dietary inclusion of 3 and 6% of its meal can increase the n-3 content of the fillet by up to 3.68% [10]. Similarly, a previous study evaluated the effect of the dietary inclusion of rapeseed oil (90%) and three levels of *Durvillaea antarctica* “Cochayuyo” brown macroalgae meal (1.5, 3 and 6%), detecting, with gas chromatography, a reduction in total n-3 levels when replacing fish oil with rapeseed oil and a partial recovery of these levels when adding the macroalgae meal [11].

Lipidome studies and massive and systematic analysis of lipid species present in cells, tissues and food have been performed, complemented by different techniques of lipid-phase extraction, mass spectrometry, bioinformatics analysis and data mining, tools that are easily accessible and available to the scientific community, allowing for the identification of more than 43,000 lipid species to date [12,13,14,15,16]. However, studies on the lipidome of foods, such as rainbow trout fillet, are scarce. So far, the effect of experimental diets on possible changes in the lipidome of this species is recent. A lipidome analysis with liquid chromatography–mass spectrometry (LC-MS/MS) could possibly provide more information on how the different lipids that make up trout muscle are remodeled as a function of dietary changes. Therefore, the aim of this study was to evaluate the effect of rapeseed oil and cochayuyo meal on the juvenile rainbow trout (*Oncorhynchus mykiss*) muscle lipidome through liquid chromatography–mass spectrometry (LC-MS/MS).

## 2. Materials and Methods

### 2.1. Animal Handling and Diets

The animals were raised and slaughtered according to the Chilean Animal Protection Law (N°20.380). The procedures were approved and supervised by the Scientific Ethics Committee of the Universidad de La Frontera (N°2319) and the Universidad Católica de Temuco.

Three hundred seventy-five rainbow trout (17.6 ± 0.46 g) were randomly grouped into five groups. The diets were developed as described by Bell et al. [10] and Dantagnan et al. [17]. The diets contained fish meal (49.5%), fish oil (13.1%), blood meal (5.0%), gluten (5.0%), cellulose (5.4%), minerals and other ingredients. Fish oil was partially replaced by rapeseed oil (90%), and cellulose was replaced by cochayuyo meal at three levels (1.5, 3 and 6%) to balance all diets. All diets used in this study were isoproteic (~47%) and isolipidic (~16%). The experimental design and chemical composition of the diets used in this study were described in detail by Quiñones et al. [11]. The first group of fish was considered a control group, and the other four groups corresponded to rainbow trout fed experimental diets, as detailed in Table 1. After 90 days, the fish were anaesthetized and sacrificed, and random dorsal muscle samples were extracted in triplicate from each group and stored at −80 °C until further analysis.

### 2.2. Extraction of the Lipid Phase from the Fillet

From each group three fish were removed completely at random and 150 mg of dorsal muscle was removed from each with a scalpel and placed in a microcentrifuge tube. Subsequently, 300 µL of cold methanol (LC-MS-grade, Fisher Chemicals, Hampton, NH, USA) was added to each tube, and the sample was homogenized using a polypropylene plastic mortar. Next, 1 mL of cold methyl tert-butyl ether (MTBE) (HPLC Plus grade, Sigma Aldrich, Saint Louis MO, USA) was added to each sample. The tubes were then shaken for 1 h at room temperature. Afterward, 250 µL of water (LC-MS-grade, Fisher Chemicals, Hampton, NH, USA) was added and kept for 5 min at room temperature for phase separation. The samples were then centrifuged at 13,000× *g* for 7 min. The upper lipid phase was recovered in a sterile tube and placed in a speed vac for 40 min based on a procedure described by Matyash et al. [18]. Finally, the samples were transferred to an HPLC vial and placed in the UHPLC Autosampler at 4 °C.

### 2.3. LC-MS Data Acquisition

Lipidome analysis was carried out through liquid chromatography–mass spectrometry (LC-MS). A compact quadrupole time-of-flight (QTOF) mass spectrometer coupled to an Elute Ultra High-Performance Liquid Chromatograph (UHPLC) (Bruker, Billerica, MA, USA) was used. Chromatographic separation was performed with an Intensity Solo UHPLC C18, 2 × 100 mm column (Bruker, Billerica, MA, USA) using a gradient with solvent A (60% acetonitrile, 0.1% acetic acid, 10 mM ammonium acetate) and solvent B (90% isopropyl alcohol, 10% ceric ammonium nitrate, 0.1% acetic acid, 10 mM ammonium acetate). A 26 min gradient was created starting with 10% B for 3 min, reaching 90% B at 16 min, held for 5 min and then reversed to 10% B for another 5 min. The ESI tuning mixture was used for system calibration with the same gradient for mass spectrometry acquisition, obtaining sample data in positive and negative ionization modes. The source parameters were 4 kV voltage, 1.8 Bar nebulizer gas, 220 °C drying gas and 9 L/min gas flow rate. Data acquisition was carried out with a sweep rate of 4 Hz and a cycle time of 2 s.

### 2.4. Data Analysis

Metaboscape 4.0 software (Bruker, Billerica, MA, USA) was used for data analysis. Data were recalibrated and aligned with feature detection for intensities above 1000 counts and at least 8 MS1 spectra per peak. Spectral libraries from the MassBank of NorthAmerica (Experimental Spectra and LipidBlast, Los Angeles, CA, USA) were used for metabolite identification. Data derived from lipidome analysis were initially processed using Microsoft Excel spreadsheet software (Microsoft Corporation, Redmond, WA, USA). Ion counts were log-transformed in base 2 for ease of analysis. The data were tabulated and subjected to a Levene test of homogeneity of variance, obtaining a value *p* > 0.05, so nonparametric statistical tests were used for this study. To determine the overall effect of the experimental diets on fish muscle lipidome, we performed a Kruskal–Wallis test with a *p*-value of *p* > 0.05 significance. Comparison of lipid counts corresponding to each diet evaluated concerning the control group was performed using the Mann–Whitney U test with *p* < 0.05. All analyses were carried out with the SPSS

Statistics^®^ 23 software (IBM, Armonk, NY, USA). Visualization of lipid counts in all conditions was performed by generating heat maps with the use of Data Mining Orange software version 3-3.30.1 (Demšar et al., 2013), where a K-means analysis with cluster = 50 was used for the specific lipid analysis.

## 3. Results and Discussion

This study evaluated the muscle lipidome of juvenile rainbow trout (*Oncorhynchus mykiss*) fed 90% rapeseed oil (*Brassica napus*) and three levels of cochayuyo (*Durvillaea antarctica*) meal (1.5, 3 and 6%), with an emphasis on the variation in the levels of lipid species composed of highly unsaturated fatty acids (HUFA), which, in later stages of growth of this species, could play a crucial role in the fish nutritional value. The effect of these experimental diets on muscle quality and fatty acid profile was previously reported and analyzed using gas chromatography (GC/FID) [11]. However, this technique is less sensitive and informative than liquid chromatography with mass spectrometry (LC-MS/MS). In this context, the present analysis detected 92 lipid species in the negative scan mode and 236 species in the positive scan mode (range: 171-944 *m*/*z*) (Supplementary Data).

The most abundant lipids identified in the negative mode corresponded to subclasses such as glycerophosphoethanolamines (PE: 35.87%), fatty esters (FAHFA: 25%), fatty acids (FA: 14.13%) and glycerophosphoglycerols (PG: 9.78%), and ceramides, octadecanoids, quinones and steroid conjugates were also identified, while in the positive scan mode, mainly glycerophospholines (69.2%), glycerophosphophosphoglycerolamines (14.77%) and phospho-sphingolipids (7.59%) were identified (Supplementary Data). In smaller proportions, ceramides (2.11%), diglycerides (1.27%), triglycerides (0.84%), fatty amides (0.84%), sphingoid bases (0.42%), isoprenoids (0.42%) and conjugated fatty acids (0.42%) were detected (Supplementary Data).

Although there is sufficient evidence demonstrating the role of lipids in fish’s nutritional value, there are very few studies on the lipidome in this kind of food [19]. There are some studies on rainbow trout, but they do not focus on evaluating the dietary effect on the muscle lipidome of this species. The impact of different vegetable oils on the lipidome (LC-MS) of tilapia fillet (*Oreochromis niloticus*) was reported by Liu et al. [20], who observed variations in the total amount of lipid subclasses and different distributions of fatty acids bound to glycerol, detecting mainly triglycerides, phosphatidylcholine and phosphatidylethanolamines, among others. Likewise, a recent study determined the effect of the inclusion of palm and flaxseed oil in seawater and freshwater conditions in the diet on lipidome remodeling (UPC2-MS/MS, positive-mode scanning) of *Salmo salar* (200 g in weight) muscle, showing that the main lipids present in this species correspond to triacylglycerols, phosphatidylcholine and phosphatidylethanolamine. It was observed that seawater allows for more active lipidome remodeling than freshwater-reared fish, mainly due to the more active lipid metabolism in the smoltification process [21].

In the present study, unlike previously described, triglycerides were low in abundance. Only two species were observed: TAG 16:0-18:1-22:0 (*m/z* 934.85) and TG 18:1-18:1-18:1-18:1 (*m*/*z* 902.79). The latter showed almost 20 ion counts, a high value in relation to the other species detected. These differences may be related to the lipid metabolism characteristic of freshwater-farmed rainbow trout and their life cycle since the muscle tissue analyzed in this study corresponded to dorsal muscle from juvenile fish weighing approximately 86.73 ± 2.6 g, a reduced size compared to other studies, such as that of Yu et al. [22], who carried out lipidomic studies on 400 g trout fillets. The age of the fish modulates the total fat content, which increases with the size of the fish in rainbow trout. Additionally, muscle type has a significant effect on lipid deposition as visceral fat content shows the same pattern of evolution as white muscle with the age of the fish, while dorsal adipose tissue reaches a constant level once trout have reached a certain age [23]. This could indicate a rapid metabolization of triglyceride with a higher growth rate since, in finfish, triglycerides are rapidly β-oxidized at the mitochondrial level for energy. It has been observed that trout regulate triglyceride content in tissues through the expression of lipid metabolism genes such as the Fatty Acid Synthase gene (FASN) and a transcription factor called Sterol regulatory element-binding transcription factor 1 (SREBP1), which is responsible for the de novo synthesis of these lipid species at the liver level [24].

**Figure 1:**
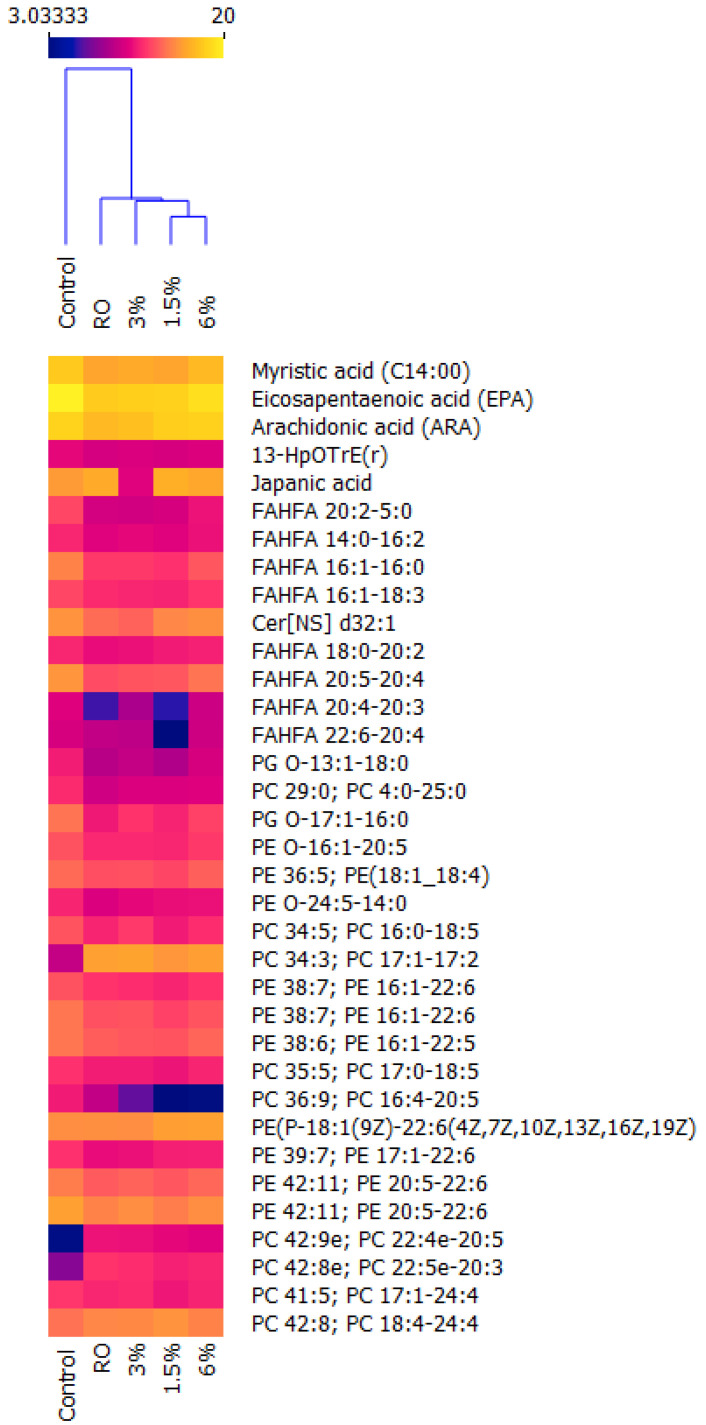
Effect (*p* < 0.05%) of rapeseed oil (*Brassica napus*) and cochayuyo (*Durvillaea antarctica*) meal on the lipidome of juvenile rainbow trout (*Oncorhynchus mykiss*) muscle analyzed with LC-MS/MS. RO: rapeseed oil; 1.5%:1.5% cochayuyo meal; 3%:3% cochayuyo meal; 6%:6% cochayuyo meal.

A Kruskal–Wallis test indicated that lipid species’ count levels were significantly affected by all the diets, which was best plotted using a hierarchical cluster and heat map (Figure 1). It can be seen that the lipidome of the groups subjected to experimental diets differed significantly from that of the control group, containing important lipids such as EPA and ARA and reducing their levels in comparison to the control group, which is in agreement with the results reported by Quiñones et al. [11]. This can also be seen in other lipid species, such as FAHFA, but they seem to stabilize with the inclusion of 6% cochayuyo meal. On the contrary, many phospholipid species are increased due to the diets. Mainly phospholipids are made up of polyunsaturated fatty acids of the n-3, 4, 5 and 6 types, which confirms the plasticity of the lipidome of rainbow trout muscle. In fish, de novo lipogenesis occurs mainly in the liver and is negligible in muscle and adipose tissue. However, like the liver, lipid uptake in muscle and adipose tissue is mediated by lipoprotein lipase (LPL), the differentiation cluster (CD36), a family of fatty acid transporter proteins (FATP1-6), plasma-membrane-associated fatty-acid-binding proteins (FABPpm), the LDL receptor (LDLR), LDL-receptor-related protein-1 (LRP1) and scavenger receptor class B type I (SRBI) [25]. In fish such as rainbow trout, these fatty acids can be acquired directly through the diet or be synthesized de novo by a lipogenic pathway that includes FASN, SCD, Elov enzymes, transport proteins (VLDL, LDL, HDL, etc.) and lipolytic enzymes (Phospholipase A and lipases), among others. Fatty acid synthase (FAS) is a key enzyme involved in lipogenesis that catalyzes the synthesis of long chain fatty acids, mainly by catalyzing acetyl coenzyme A and malonyl coenzyme A into the final product, palmitate, which is subsequently esterified into TAG and stored in adipose tissue, and its expression is affected by dietary fish oil restriction in salmon and trout species [23,26].

Furthermore, Stearoyl-CoA desaturase (SCD) synthesizes oleate necessary for triglycerides and other lipids’ biosynthesis. It plays a critical role in transforming saturated fatty acids into unsaturated fatty acids by inducing the first double bond between the C9 and C10 from palmitic acid to palmitoleic acid and stearic acid to oleic acid. The Elongases of very long chain fatty acids (ELOV) are some of the major enzymes in the pathways for the elongation of C18 PUFAs to C20/22 unsaturated fatty acids [27].

**Figure 2:**
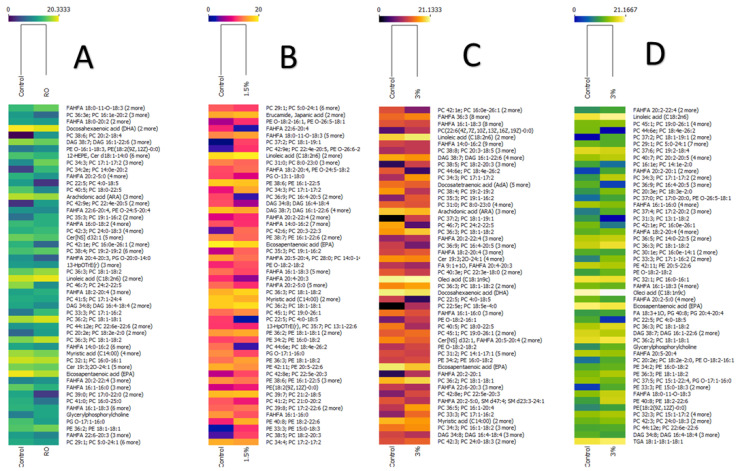
A significant effect (*p* < 0.05) of rapeseed oil (*Brassica napus*) and cochayuyo *(Duvillaea antarctica)* meal on lipid species of juvenile rainbow trout (*Oncorhynchus mykiss)* muscle was determined with the Mann–Whitney U test. (**A**) 90% rapeseed oil (RO); (**B**) 90% of RO+1.5% cochayuyo meal; (**C**) 90% of RO+3% cochayuyo meal and (**D**) 90% of RO+6% cochayuyo meal. Lipids are presented in relation to their ion count abundance with respect to the control group.

A Mann–Whitney U test allowed us to detect how count levels varied between the experimental diets and the control group (Figure 2). It was observed that the lipidome and ion counts of the different lipid species changed in relation to diet content. The fatty acid composition of rainbow trout fillet has been widely described [9,28]. However, in this study, lipidome analysis allowed for the detection of a wide variety of other lipid species. Specifically, free fatty acids were detected, which corresponded mainly to fatty acids of nutritional interest, such as myristic acid (C14:0, *m*/*z* 272.2), palmitic acid (C16:0, *m*/*z* 255.2), oleic acid (C18:1 *m*/*z* 281.3), linoleic acid (C18:2, *m*/*z* 279.2), arachidonic acid (ARA, *m*/*z* 303.2), eicosapentaenoic acid (EPA, *m*/*z* 301.2), eicosatrienoic acid (AdA, *m*/*z* 331.3) and docosahexaenoic acid (DHA, *m*/*z* 327.2). Myristic acid, EPA and DHA were the fatty acids that decreased in all experimental groups. In contrast, linoleic acid increased, probably due to the content of this fatty acid in rapeseed oil, as reported by Quiñones et al. [11].

Less studied lipid species were also identified, among them fatty acid esters of fatty hydroxy acids (FAHFA). We observed some species consisting of n-3 and n-6 fatty acids, such as FAHFA 16:1-18:3 (*m*/*z* 655.5), FAHFA 20:5-20:4 (*m*/*z* 603.45), FAHFA 20:4-20:3 (*m*/*z* 607.48) and FAHFA 22:6-20:4 (*m*/*z* 629.46), which had significantly reduced levels in all groups (Figure 1). FAHFA corresponds to a lipid family found in animal tissues and is mainly constituted by two fatty acids and a hydroxyl group representing a large reservoir of neutral lipids released by lipolytic enzymes [29]. In humans, this type of lipid can be synthesized endogenously but can also be acquired through ingesting animal products, and has recently been considered to be a bioactive lipid with anti-inflammatory and antidiabetic effects [30,31]. However, their presence in rainbow trout muscle has not been reported so far.

In the present study, we identified a great variety of phospholipids, such as phosphatidylcholine (PC), phosphatidylethanolamine (PE), phosphatidylserine (PS) and phosphatidylinositol (PI), which correspond to polar lipids present in all biological membranes (cellular, mitochondrial, peroxisomal, etc.), mainly fulfilling a structural role, since they are major cellular components and to date are considered as primordial as nucleic acids for the formation of new cells [32]. McMurray and Magee, [33] described the factors (long-chain and alkyl fatty alcohols, triose phosphates, dihydroxyacetone phosphate) and cofactors (ATP, CoA and Mg^2+^) involved in phospholipid assembly, which, in general, can be found in all animal tissues, including fish. In the present investigation, all phospholipid subclasses (PC, PE, PI and PS) were identified in rainbow trout muscle and are produced by choline esterification, ethanolamine or serine, and inositol in the phosphate group by endogenous processes occurring mainly in the endoplasmic reticulum and Golgi apparatus. In contrast, other lipid species, such as cardiolipins and phosphatidylglycerol formation, occurs in the mitochondria and peroxisomes (plasmalogens: PlmePEtn and PlmePCho), respectively [34,35]. In fish, in the formation of phospholipids, it has been observed that saturated fatty acids (SFA) and monounsaturated fatty acids (MUFA) are esterified at the sn-1 position of phosphoglycerides. In contrast, PUFA is preferentially esterified at the sn-2 position. However, there are many exceptions, such as di-docosahexaenoyl phosphoglycerides, which are abundant in the fish retina, specifically in the outer membrane segments of the rods [35]. Still, it is also possible to find them in adipose and muscle tissue. In the present study, it was possible to observe a great variety of phospholipids consisting of sn-1 PUFA/sn-2 PUFA, such as PC 20:2-20:5 (*m*/*z* 832.56) PS 22:6-22:6 (*m*/*z* 880.49) and PC 22:6e-22:6 (*m*/*z* 864.59), among others. In a recent lipidomic study performed by HILIC/MS (negative- and positive-mode scan), it was determined that the most abundant phospholipid species in 400 g rainbow trout fillet corresponds to PC 38:6 [22], which is consistent with the results of the present study, which observed that the ion m/z 806.55 (PC 38:6; PC 16:0-22) was the most abundant species in all groups (Supplementary Data). Another study [16] detected that *m*/*z* 747.50, 771.49 and 863.55 ions could be candidates for use as biomarkers for discrimination of salmon and rainbow trout. Similarly, Yu et al. [22] compared the lipidome of rainbow trout fillet with Salmo salar and Oncorhynchus tshawytscha, identifying *m*/*z* 802.8 (PC 34:2) and *m*/*z* 834.8 (PC 36:0) ions as possible markers to distinguish between these species, which can share up to 90% of their lipidome.

Some studies carried out on fish show that the esterification of fatty acids in the sn-1/2 positions varies depending on the species and living conditions, observing that the abundance of phospholipids in the muscle of Salmo salar was lower in fish reared in seawater compared to freshwater fish, except PC and PE 44:12, which were present in greater abundance in the latter group [21], which is consistent with the results of this study. Additionally, Song et al. [16] determined by REIMS (negative scan mode) that the main n-7 lipid species detected in Salmo salar fillet correspond to phospholipids such as PC 16:1-18:1, PC 16:1-20: 2, PE 16:1-18:1 and PE 16:1-18:0, unlike those of other species, such as tuna, butterfish and shrimp, among other organisms. In this context, n-7 lipids such as PE O-16:1-18:3, PE O-16:1-20:5, PC 16:0-16:1, PC 16:1-16:1, PC16:1e/20:2 and PC16:1-16:4 were detected in this study. However, only PE 16:1-22:6 (*m*/*z* 762.49) and PE O-16:1-20:5 (*m*/*z* 720.51) count levels were significantly reduced in all treatments. DAG 16:1-22:6 (*m*/*z* 656.50) was only reduced with inclusion levels of cochayuyo. On the other hand, PC 16:0-16:1 and PC 16:1e/20:2 levels were significantly reduced with the rapeseed oil treatment. In addition, 1.5% cochayuyo meal increased the level of PE O-18:2-16:1, 3% cochayuyo meal reduced the level of PC 16:0-16:1 counts, and 6% cochayuyo meal reduced the level of PE 16:1-22:6 counts (Figure 1 and Figure 2).

Some research indicates that rapeseed oil in the diet of carnivorous fish is a viable alternative lipid source, as its triglycerides and phospholipids have moderate levels of C18:2 and C18:3, and high levels of C18:1, which makes it an excellent source of energy as a substrate for β-oxidation [36]. In addition, the C18:2/C18:3 ratio of this oil is 2:1, which has no detrimental effect on fish health, and growth has been detected [17]. Previous studies have demonstrated the effect of rapeseed oil on the fatty acid profile of trout muscle [7,9]. The results show a reduction in total EPA and DHA content and an increase in C18:2, which is consistent with the levels detected in the present study (Figure 2). However, in contrast to previous studies, our analysis detected that dietary inclusion of rapeseed oil and low levels of cochayuyo meal (1.5%) did not modify the levels of C18:1 in its free form. Still, it was observed that this type of diet had a significant effect on the increased counts of TAG C18:1-C18:1-C18:1 (*m*/*z* 902.79) and phospholipids PC 18:1-18:1 and PE 18:1-18:1. This could indicate that free fatty acids from the diet are rapidly metabolized and could be utilized by transporter routes for storage as triglycerides or included in cell membranes.

This study allowed us to detect in which lipid species the free fatty acids were distributed and their abundance when feeding fish the different diets. In this context, about 100 lipids were identified that presented variations in their count level due to the experimental diets, mainly showing an increase in the counts of phospholipids constituted by medium- and long-chain fatty acids in all treatments. Thus, the composition and level counts of the phospholipids detected can be attributed to the high levels of C18:1 present in the diets used (>40%) [11], which could be directly correlated with the lipid species, with high levels of PE 18:1-18:1 and PC 18:1-18:2 being observed in all experimental groups compared to the control group.

An in vitro study demonstrated that rainbow trout could rapidly adapt their lipid metabolism through simultaneous elongations and desaturations of fatty acids in response to dietary inclusion of vegetable oils, modifying the expression levels of genes such as Peroxisome-Proliferator-Activated Receptor Gamma (PPARγ) and Fatty acid transport protein 1 (FATP1) [37]. In the present study, it was observed that for all diets, the levels of PE 15:0-18:3 increased almost 4 times when compared with the control group. In mammals, it has been observed that the phospholipid configuration sn-1 SFA/sn-2 PUFA is a mechanism of action of the cell to ensure membrane fluidity, as SFA renders the membrane more rigid and PUFA much more fluid [38]. The substantial increase in PE 15:0-18:3 levels may possibly be a mechanism to balance cell membrane viscosity in the face of the high levels of MUFA and PUFA present in the experimental diets [11]. However, in the current literature, reports of the presence or importance of this lipid in fish muscle are scarce, so in future studies, these level changes could be better addressed.

In the present investigation, it was further observed that the main changes occurred in lipids that presented sn-1 MUFA and sn-2 PUFA configurations of medium chains and long chains such as PE O-26:6-20:4 (*m*/*z* 615.54), PC 22:4e-20:5 (*m*/*z* 842.61), PC 14:0e-20:2 (*m*/*z* 744.57) and PC 18:2-20:3 (*m*/*z* 808.56), among others. However, phospholipids such as PC 4:0-18:5 (*m*/*z* 584.31), PE 16:1-22:6 8 (*m*/*z* 762.49), PC 17:0-22:0 (*m*/*z* 832.66), PC 16:4-20:5 (*m*/*z* 772.54) and PC 18:4e-26:2 (*m*/*z* 876.69) showed more lower levels in all experimental groups.

Lipids such as diacylglycerols, glycerophospholines and phosphingolipids had reduced level counts in all groups formed mainly by combinations of SFA with from 4 to 24 carbons (PC 4:0-25:0, PC 14:0-14:0, PC 14:0e-16:0, PC 15:0-15:0 and PC 18:0e-24:0, among others) and MUFA combined with PUFA fatty acids (PE 20:5-22:6, PC 15:1-22:4, SM d23:3-24:1 and DAG 16:1-22:6, among others) (Figure 2), but an increased level count of a glycerophosphocholine species composed of eicosadienoic acid and EPA (PC 20:2-20:5 and PC 22:4e-20:5) was also observed, whereas reduced levels of PS 22:5-22:6 were detected in all the experimental groups except for the group fed with 6% cochayuyo meal.

Fatty acids such as C18:2 and C18:3 are found in abundance in rapeseed oil, and because the lipid metabolism of trout is very active, they can rapidly be deposited on cell membranes, which facilitates membrane fluidity and would allow trout to tolerate and inhabit cold temperature waters [39]. Cold-water fishes have several mechanisms, including lipogenic enzymes, that enable the reuse of fatty acids as substrates to produce new long-chain fatty acids such as C24:5 and DHA. In teleost fish, C24:5 is considered an elongation of EPA, which is mediated by the enzyme Elongation of very long chain fatty acids-4 (Elovl4), and DHA is vital for trout metabolism, increasing membrane fluidity, which allows trout to tolerate low temperatures, and consequently contributing to the nutritional improvement of the fillet [40,41]. The increased levels of phospholipids consisting of ARA, EPA and DHA are the result of the metabolization of C18:2 and C18:3 fatty acids from rapeseed oil that served as a substrate for delta Fatty acyl desaturases (Fad D5 and D6) enzymes, which introduce double bonds in the carbon chain, and an Elovl enzyme adds 2 carbons to the chain, resulting in C20:4 (ARA). The synthesis of EPA from C18:3 requires the same lipogenic scheme as ARA, but the synthesis of DHA occurs by two additional elongation steps, a Δ6 desaturation and a peroxisomal-chain-shortening step [42].

Macroalgae are a very abundant bioresource on our planet and can be found in various latitudes. There are three taxonomic groups represented by the term “macroalgae”: Rhodophyta (red), Chlorophyta (green) and Phaeophyta (brown), as well as cochayuyo (*Durvillaea antarctica*). This has stimulated the study of these groups and the characterization of metabolites (proteins, lipids, mineral salts and antioxidants, among others) that can be considered functional in many fields, but recently, they have been considered as potential functional additives for aquafeeds [43,44].

Because the fatty acid profile in the body of fish reflects the fatty acid profile of their diet, several reports indicate that macroalgae supplementation could increase the amount of n-3 in the muscle of carnivorous fish and could counteract the total reduction in n-3 in trout fillet, as a consequence of the dietary inclusion of vegetable oils, such as rapeseed oil [10,45]. For example, the dietary inclusion of 10% *Ulva lactuca* resulted in a significant increase in trout muscle Docosapentaenoic acid (22:5n-3; DPA) levels at the end of the feeding period [46]. On the contrary, in this study, the count levels of n-3 fatty acids and lipid species such as PC 22:5e-20:3 were reduced by all experimental groups. Additionally, Bruni et al. [45] reported a commercial macroalgae extract in a plant-protein-rich diet with diminished saturated fatty acids fed to trout fillets, which is consistent with our study, mainly with the reduction in myristic acid, FAHFA and phospholipids containing saturated fatty acids with from 4 to 18 carbons.

In the heat maps of the Mann–Whitney U Test, it can be observed that the increase in the amount of cochayuyo meal lead to an increase in the number of phospholipid species, increasing their count levels in relation to the control group (Figure 2). One of the main increased species corresponded to FAHFA, particularly those constituted by from 18 to 20 carbons, and only FAHFA 18:3-18:2 (*m*/*z*: 555.45) and FAHFA 20:2-20:1 (*m*/*z*: 615.54) increased their counts in the groups fed with 3 and 6% cochayuyo meal. In addition, free oleic acid also increased, but only in the latter two groups. Likewise, the number of phospholipids (PC, PE and PG) increased when cochayuyo was included in the diet. We can appreciate that, unlike the diet that only had rapeseed oil, cochayuyo seems to provide long-chain phospholipids composed mainly of polyunsaturated fatty acids that vary in chain length (18-26 carbons) and the amount of unsaturation, such as PC 15:1-17:2 (*m*/*z*: 728.54), PC 17:2-22:6 (*m*/*z*: 816.57) and PC 18:4e-22:5 (*m*/*z*: 814.55), among many others. The rest of the lipid species identified were significantly reduced by the diets, which affected free fatty acids, FAHFA and phospholipids made up of n-3 and -6 fatty acids. Cochayuyo possesses high levels of linoleic acid (C18:2) [43]. It is possible that PC 18:4e-22:5 and PC 18:4-24:4, consisting of stearidonic acid (C18:4), and elongation of ARA (C24:4) correspond to n-3-derived lipids and may explain the high levels of total n-3 in the fatty acid profile reported in previous studies [11,47].

It has been suggested that seaweeds are excellent sources of n-3 fatty acids, potentially enhancing the fish muscle lipid profile compared to the proportion of n-3 fatty acid concentration [44]. Some studies, such as those reported by Safavi et al. [48], show that different numbers of polysaccharides of *Ulva intestinalis* and *Gracilaria persica* in diets had no significant effect on muscle fatty acids of the rainbow trout fillet. This could be due to polysaccharides in seaweeds, as cochayuyo can stimulate lipid metabolism. The body catabolizes fatty acids as the primary energy expenditure, explaining the increase in phospholipid species detected in this study [49,50].

The different inclusion levels of cochayuyo meal used in this study (1.5, 3 and 6%) were not sufficient to cause more remarkable changes in the lipidome; because rainbow trout are carnivorous fish, it is not possible to incorporate levels higher than 10% macroalgae supplementation into their diet, as this may limit fish growth [46,49]. Cochayuyo also has high levels of n-3 and n-6, but the total lipids of this species are very low compared to other macroalgae species [43]. It is also possible that the contribution of the cochayuyo may have been masked by the fatty acids from the rapeseed oil. However, according to the results of this study and what has been reported in the literature, cochayuyo is a brown macroalgae containing multiple functional nutrients, such as β-glucan, that may affect other aspects of the production cycle of carnivorous fish, mainly health and disease resistance, and the use of other macroalgae with a higher total lipid content could perhaps generate a more significant effect on trout muscle lipidome [51].

## 4. Conclusions

In this study, the effect of experimental diets on the lipidome of rainbow trout muscle was analyzed by LC-M/MS, demonstrating that the lipidomic is a tool that can be used to explore and characterize, more specifically, the lipids and their composition. Furthermore, the results reinforce the hypothesis concerning the plasticity of the lipidome of species such as rainbow trout in response to dietary changes. Rapeseed oil drastically modifies the lipidome of rainbow trout muscle since it reduces the levels of lipid species such as FAHFA, which has not been previously described in rainbow trout and is made up of unsaturated fatty acids, as well as phospholipids such as PC, PE and PG, which is consistent with what has been reported in other studies. However, it seems that the contribution of lipids from cochayuyo meal was masked by rapeseed oil; as the dietary inclusion increased, the number of unsaturated phospholipids rose, which increased their levels. The nutrient content of the macroalgae possibly modifies lipid metabolism, but further studies are needed to validate this. The use of seaweed species with a higher total lipid content could have an even more significant impact on the muscle lipidome of commercial fish such as trout.

## Figures and Tables

**Table 1:** Dietary ingredients for the experimental feeding of rainbow trout (*Oncorhynchus mykiss*).

Ingredients % DM	Control	RO	1.5%	3%	6%
Fish meal	100	100	100	100	100
Fish oil	100	10	10	10	10
*Brassica napus* oil	0	90	90	90	90
*D. antarctica* meal	0	0	1.5	3	6
Cellulose	5.4	5.4	4.1	2.7	0

RO: *Brassica nappus* oil. The diets’ nutritional composition and fatty acid profile were reported in Quiñones et al. (2021).

## Supplementary Materials

The following are available online at https://www.mdpi.com/article/10.3390/biom12060805/s1, Table S1: Lipidome of juvenile trout muscle analysed by LC-MS/MS.
